# County Has No Right to Discharge Physician without Cause

**Published:** 1898-10

**Authors:** 


### County has no Right to Discharge Physician Without Cause.

The first of several questions deemed important enough to be specially certified up to the Supreme Court of Texas, in the case of Galveston County vs. Ducie, was as to whether the commissioners' court had authority to appoint a county physician, at a stated salary, to perform duties comprising the giving of medical attention to the prisoners at the jail, both the criminals and the pauper insane and county paupers at the poor farm, and any one sick within the jurisdiction of the county, confined as a prisoner, pauper or lunatic, and to attend inquests whenever anybody was found dead. The answer of the Supreme Court, handed down May 9, 1898, is that the commissioners' court of Galveston county was authorized to make the contract for medical services to be rendered to paupers and prisoners for whose care and support the county was required to provide, but that it had no authority to make the contract for the physician's services at inquests, and that to that extent the contract made was not binding upon the county. The explanation of this is that counties are not liable for the services of medical men at inquests which may be held under the provisions of the Texas Code of Criminal Procedure. Yet, as already suggested, the court holds that, although the stipulation in the contract for the physician's services at inquests was ultra vires, or beyond the power of the commissioner's court to contract, it did not render the agreement invalid for that part which the county had the power to contract for. And it insists that, so far as the commissioners' court had authority to make the contract referred to, the county was bound to the same extent an individual would have been, and it had no right to discharge the physician without cause. If wrongfully discharged, he would be entitled to recover the amount contracted to be paid him, less so much as attendance upon all inquests in the county would be reasonably worth, and less any additional sum the evidence might show that he could make in the practice of his profession by being relieved of attending to the county cases. But the contract in question, not contemplating that the services to be rendered for the county would occupy all of the physician's time, but that he might give what spare time he had to private practice, the court holds that, when wrongfully discharged he would not be obliged to seek other employment, to reduce the damages against the county, because that would not be consistent with his regular business, some weight also being attached to the fact that it did not appear that there was any other business of like character in which he could have obtained employment.—*Journal of the American Medical Association.*

